# Decreased Spontaneous Electrical Activity and Acetylcholine at Myofascial Trigger Spots after Dry Needling Treatment: A Pilot Study

**DOI:** 10.1155/2017/3938191

**Published:** 2017-05-16

**Authors:** Qing-Guang Liu, Lin Liu, Qiang-Min Huang, Thi-Tham Nguyen, Yan-Tao Ma, Jia-Min Zhao

**Affiliations:** Department of Sport Rehabilitation, School of Kinesiology, Shanghai University of Sport, Shanghai, China

## Abstract

**Objective:**

The aims of this study are to investigate the changes in spontaneous electrical activities (SEAs) and in acetylcholine (ACh), acetylcholine receptor (AChR), and acetylcholine esterase (AChE) levels after dry needling at myofascial trigger spots in model rats.

**Materials and Methods:**

Forty-eight male Sprague-Dawley rats were divided into four groups. Thirty-six rats were assigned to three model groups, which underwent MTrSs modeling intervention. Twelve rats were assigned to the blank control (BC) group. After model construction, the 36 model rats were randomly subdivided into three groups according to treatment: MTrSs model control (MC) and two dry needling groups. One dry needling group received puncturing at MTrSs (DN-M), whereas the other underwent puncturing at non-MTrSs (DN-nM). Dry needling treatment will last for two weeks, once a week. SEAs and ACh, AChR, and AChE levels were measured after one-week rest of dry needling treatment.

**Results:**

The amplitudes and frequencies of endplate noise (EPN) and endplate spike (EPS) significantly decreased after dry needling treatment in the DN-M group. Moreover, ACh and AChR levels significantly decreased, whereas AChE significantly increased after dry needling treatment in the DN-M group.

**Conclusion:**

Dry needling at the exact MTrSs is more effective than dry needling at non-MTrSs.

## 1. Introduction

Myofascial trigger points (MTrPs) are hyperirritable spots in a palpably taut band of skeletal muscle in humans. Clinical symptoms of MTrPs include spontaneous pain and referred pain. Puncturing or pressing MTrPs produces local twitch responses (LTRs). The LTR is a palpable or visible brisk contraction of the muscle fibers in or around the taut band [1]. Some patients describe this as a little electrical shock; others feel it more like a cramping sensation. It is a good and desirable reaction to elicit local twitch responses during treatment [2].

Histological and electrophysiological studies of myofascial trigger spots (MTrSs), which are similar to human MTrPs in many aspects [3, 4], have revealed pathological changes with abnormal contracture knots in muscle fibers and electromyographic (EMG) features of spontaneous electrical activity (SEA) [1, 5, 6]. Abnormal contracture knots may be initiated by a synaptic dysfunction at the motor endplate with the excessive acetylcholine (ACh) [7] release and changes in ACh receptor (AChR) [8] or acetylcholinesterase (AChE) [9] activities. Needling EMG studies have shown that SEA is a combination of two bioelectrical signals, such as endplate noise (EPN, continuous, low amplitude, and noise-like action potentials at 10–80 microvolts) and endplate spikes (EPS, intermittent large amplitude spikes at 100–600 microvolts) [10], that may result from excessive ACh release in the motor endplate [11].

MTrPs can be inactivated with a series of treatments, such as transcutaneous electrical stimulation, ultrasound or massage, injections, acupuncture, and dry needling [12, 13]. Dry needling is widely used in clinical work and is a well-established treatment for MTrPs [14, 15]. Previous research has revealed that dry needling decreases SEA amplitudes significantly [16, 17].

In our previous study, MTrSs in a rat muscle were created by a blunt strike and eccentric exercise, which showed different changes in spontaneous electrical activities [5]. However, the changes in SEAs and biochemical substances (ACh, AChR, and AChE) after dry needling at MTrS remain unknown. The aim of this study is to investigate the physiological and biochemical effects of dry needling at MTrSs for two weeks.

## 2. Materials and Methods

### 2.1. Animals

Forty-eight male Sprague-Dawley rats (mean age 7 weeks, weighing 220–260 g) were divided into four groups. Thirty-six rats were assigned to three model groups, which underwent the MTrS modeling intervention. Twelve rats were allocated to the blank control (BC) group. The rat model groups were constructed via blunt striking injury and eccentric exercise that were applied to the left gastrocnemius muscle (GM) for 8 weeks and with a recovery period of 4 weeks. After modeling, the 36 rats were randomly subdivided into three groups: MTrSs model control (MC) group, a dry needling group for puncturing at MTrSs (DN-M), and a dry needling group for puncturing at non-MTrS (DN-nM). All rats were housed in polypropylene cages with a 12 h/12 h light-dark cycle in a temperature-controlled room (20°C–25°C) at a relative humidity of 40%–70%. Food and water were freely provided. All experiments were conducted in accordance with the ethical guidelines of the International Association for the Study of Pain in Animals [18, 19] and the regulations set by the Administration of Affairs Concerning Experimental Animals. The individual study protocol was approved by the Shanghai University of Sport Science Research Ethics Committee (permission number 2013012, license number SCXK 2007-0003).

### 2.2. Modeling Intervention

The 36 rat models were anesthetized with injections of 4 mL/kg 10% chloral hydrate into the abdominal cavity and then fixed on a board with a homemade striking device [6]. The site of the left GM was marked. The rats were hit once on the first day of every week at the marked position. The hit was completed with a stick dropped from a height of 20 cm with a kinetic energy of 2.352 J to induce muscle contusion. On the second day of each week, the rats were made to run for 90 min on a treadmill (DSPT-202, China) at a −16° downward angle and speed of 16 m/min. The rats rested for the remaining days of the week. The interventions were repeated at weekly intervals for a total of 8 weeks. The rats were then allowed to recover for 4 weeks.

### 2.3. Identification of MTrS

Prior to the administration of an anesthetic, the taut bands of the left GM were identified by grasping with the fingers and were palpated by gently rubbing [20]. The reaction (withdrawal of the lower limb, turning the head, or screaming) of the animal was observed to confirm the exact location of MTrS [21]. The identified MTrS region was marked on the skin with an indelible black marker. The animals were then anesthetized and fixed on a board as above. The fur around the left GM of rats was shaved off with an electric hair trimmer. Then, the MTrS was reconfirmed by EMG examination. The previously marked region was only remarked precisely with an indelible red marker if EMG was founded. The red-marked region of the left GM was designated for dry needling treatment and EMG studies.

### 2.4. Dry Needling of the Left GM

DN-M and DN-nM group rats were anesthetized and fixed on a board as above. Then, dry needling was performed. For the DN-M group, a needle (300 *μ*m in diameter, 2.5 mm in length, Wuxi Jiajian Medical Instrument Co., Ltd., China) was inserted into the red-marked region of the left GM and moved back and forth at different directions in fast speed to elicit as many LTRs as possible [1, 19]. For the DN-nM group, the needle was inserted into an unmarked region of the left GM (outside the marked MTrS region with nontaut band, non-MTrS) and punctured similarly. However, the direction of needling needs to change quickly if it elicited LTR, in order to avoid inserting into the MTrS region in DN-nM group. Generally, fifteen repeated punctures were performed on the left GM in both groups. The treatment was performed once per week for two weeks.

### 2.5. Examination of EMG Recording

Four groups of rats were anesthetized and fixed on a board as above. SEAs were examined when modeling was completed and one week after the dry needling treatment. SEA recordings were measured with three fine-needle electrodes (Ф0.3 mm) by EMG equipment (NeuroCare-E, NCC Medical Co., Ltd., Shanghai, China, sampling frequency at 50,000 Hz). A band with a filter was set at 20 Hz to 3000 Hz. The gain was set at 20 *μ*v/division. The sweep speed was 10 ms/division. The first electrode (ground electrode with green color) was inserted in the tail of the rat and the second electrode (reference electrode with red color) was inserted into the red-marked region. A local twitch response indicated a possible MTrS. Thus, a third electrode (recording electrode with black color) was inserted longitudinally approximately 3−5 mm away from the second electrode. MTrS was represented by SEA with an amplitude that exceeded 10 *μ*v. The examiner would then stop advancing the needle and would minimally move the needle at different directions to obtain the SEA with the highest amplitude. If the SEA could not be found, the searching needle would be moved to another site until a satisfactory SEA tracing could be obtained. The EMG of the confirmed SEA was recorded for 2 min and the peak-to-peak value within groups was calculated. EPN and EPS were distinguished at 10–80 microvolts (noise-like action potentials) and at 100–600 microvolts (intermittent large amplitude spikes) [10], respectively.

### 2.6. Enzyme-Linked Immunosorbent Assay

The GM was dissected out after the last EMG recording. Animals were sacrificed with an overdose of anesthetic before dissection. In total, 100 mg of muscle tissues from MTrSs was collected from deep red-marked sites and placed in 900 *μ*l phosphate-buffered saline (PBS; pH 7.4). The tissues were quickly fragmented with a pair of ophthalmic scissors and homogenized with an ultrasonic wave (10 s on and 10 s off, repeated 2-3 times) and a high-speed vibration device (6000 rpm, 20 s on and 10 s off, repeated 2-3 times). The tissue homogenates were centrifuged at 8,000 rpm at 4°C for 10 minutes. The supernatant was then extracted and stored at −80°C. The contents of ACh, AChR, and AChE proteins in samples were measured with ACh, AChR, and AChE ELISA kits (A21170, J15187, and G12151; Westang, Shanghai, China). ACh, AChR, and AChE were quantified in accordance with the manufacturer's ELISA protocol. Optical density (OD) was read at 450 nm within 30 min using the microplate reader (Denley Dragon Wellscan MK 3, Finland). Concentrations were calculated according to the standard curve using Ascent™ Software (version 2.6, Finland).

### 2.7. Statistical Analysis

Mean and standard deviations for EPN, EPS, ACh, AChR, and AChE levels were calculated. Two-way mixed-design analysis of variance was used to compare the differences of EPN and EPS among groups as well as before and after treatment, whereas one-way analysis of variance was used to compare the differences of ACh, AChR, and AChE levels among groups. Post hoc comparisons among groups were examined using Bonferroni's method. The confidence interval was set at 95% (*p* < 0.05) and 99% (*p* < 0.01).

## 3. Results

### 3.1. The Effects of Dry Needling MTrS on SEA

**Figure 1:**
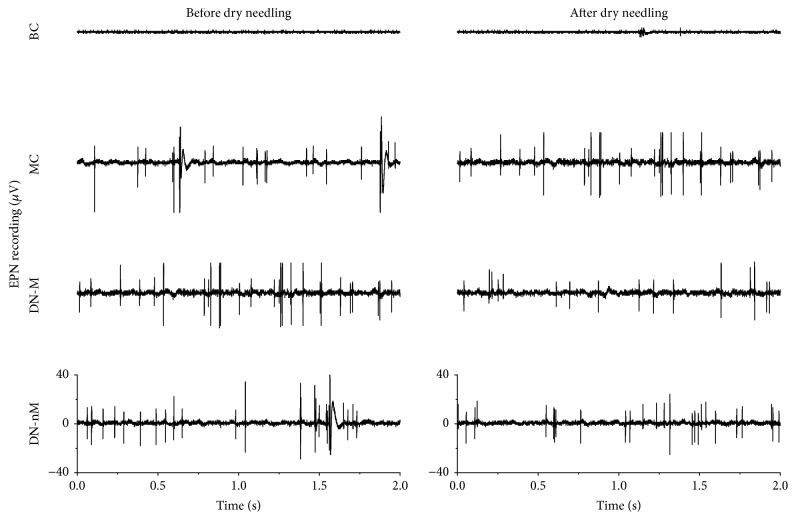
Typical endplate noise (EPN) from MTrSs of the gastrocnemius before and after dry needling in different groups. BC (blank control), MC (MTrSs control), DN-M (dry needling at MTrSs), DN-nM (dry needling at non-MTrSs), and MTrSs (myofascial trigger spots).

**Figure 2:**
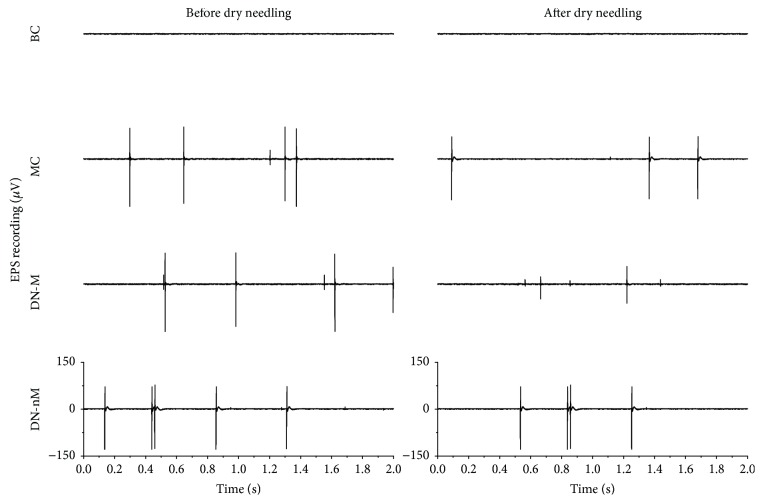
Typical endplate spikes (EPS) from MTrSs in the gastrocnemius before and after dry needling in different groups. BC (blank control), MC (MTrSs control), DN-M (dry needling at MTrSs), DN-nM (dry needling at non-MTrSs), and MTrSs (myofascial trigger spots).

Typical EMG recordings showed the EPN and EPS changes before and after intervention of all modeling groups. Only a horizontal baseline appeared in the blank control group (Figures 1 and 2), which verified that the rat model of MTrS models was successfully constructed.

Quantifying EMG revealed lower activity in both the amplitudes and frequencies of both EPN and EPS in the blank control group. By contrast, the other groups exhibited higher activities either before or after dry needling (Table 1). The activities of both amplitudes and frequencies for both EPN and EPS significantly changed before and after dry needling in the DN-M group (*p* < 0.01) (Table 1) but not in the MC group (*p* > 0.05). Moreover, the frequencies of EPN in the DN-nM group did not change. Compared with those in the MC group, the EPN and EPS of both amplitudes and frequencies of the DN-M group significantly decreased after dry needling (*p* < 0.01) (Table 1). Both the EPN and EPS in the DN-M group were significantly lower than in the DN-nM group after dry needling (*p* < 0.01) (Table 1).

### 3.2. Levels of ACh, AChR, and AChE

**Figure 3:**
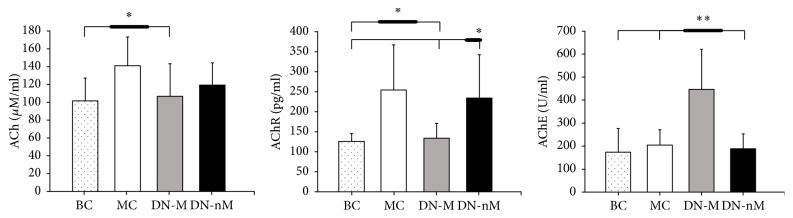
Levels of ACh, AChR, and AChE. ^*∗*^*p* < 0.05, ^*∗∗*^*p* < 0.01. BC (blank control), MC (MTrSs control), DN-M (dry needling at MTrSs), and DN-nM (dry needling at non-MTrSs).

After dry needling, the MC group had a significantly higher ACh level than the other groups (*p* < 0.05), whereas there was no difference in ACh levels among the other groups (*p* > 0.05). AChR level significantly decreased in the DN-M group compared with those in MC and DN-nM groups (*p* < 0.05) and was close to that of the BC group (*p* > 0.05). AChE concentration was significantly higher in the DN-M group than in the other groups (*p* < 0.01). Moreover, there was no difference in AChE concentration among the other groups (*p* > 0.05) (Figure 3).

## 4. Discussion

In this study, the effects of dry needling at the precise site of MTrSs, as compared with those of dry needling at non-MTrSs regions, are shown for the first time. The former reduced the amplitudes and frequencies of EPN and EPS, decreased ACh and AChR, and increased AChE. The latter did little and may not be exactly effective. The current study demonstrated that dry needling therapy could effectively improve the physiological and biochemical status of MTrSs only if it precisely punctures MTrSs.

EPN is more likely to occur at a MTrP site [7]. The electrical activities of MTrPs are definitively associated with motor endplates [22]. The EPN is an abnormal waveform pattern that is associated with an increased rate of spontaneously released ACh packets from the motor endplate [11]. In the current study, SEA was only found in the MTrS model rats. After dry needling, EPN amplitudes decreased at MTrSs compared with the pretreatment baseline. EPN amplitudes also significantly decreased after dry needling in the DN-M and DN-nM groups. The EPN amplitudes of the latter group decreased less than the former. Both the amplitudes and frequencies of EPN and EPS declined together only in the DN-M group. This result indicated that needling at the exact MTrS is effective. Despite a significant decrease in SEA amplitudes and frequencies in the DN-M group, the SEA still existed and did not completely disappear. This result suggested that dry needling cannot completely treat MTrSs with only one or two treatments but can reduce the amount of discharges from MTrS. Therefore, pain that originates from the MTrS may require repeated needling to be treated.

The motor endplate hypothesis considers that excessive ACh production causes the characteristic SEA of MTrSs. Injecting botulinum toxin into MTrPs can reduce EPN by blocking the release of ACh into the synaptic cleft, thus supporting the motor endplate hypothesis [20]. Similarly, the MC group had a higher ACh concentration than the BC group, which means excessive release of ACh into the MTrS site. Therefore, high ACh concentrations induce local excitatory discharges in skeletal muscle fibers, which create contracture knots.

AChR levels increased with ACh levels in the MC group. The relative excess of AChR occurs in numerous pathological situations [23], such as motor neuron lesions [24], muscle trauma [25], and burns [26]. Usually, the currents generated at miniature endplates depend on the rate constants of ACh-AChR binding. ACh activates AChR, which causes a muscle action potential and persistent muscle contraction [23]. The significantly decreased ACh and AChR levels in the DN-M group indicated that dry needling at MTrS exerts positive effects by decreasing both the amplitudes and frequencies of SEA, which relieves taut bands within a muscle. By contrast, AChE levels in the DN-M group significantly increased after dry needling. Therefore, dry needling may play a role in relieving the status of contracture.

The penetration of a needle through the skin can produce physiological effects via the activation of the diffuse noxious inhibitory control, a pain-suppressing system in the spinal cord [27]. In the present study, high-speed insertions were applied at different directions of MTrSs, as suggested by Hong [28]. High-speed needling at MTrP regions provides immediate and complete pain relief by exerting high-pressure stimulation, which elicits LTRs [2]. Dry needling MTrPs supports this efficient strategy by diminishing EPN if LTRs were elicited [16]. In addition, dry needling MTrSs can diminish EPN by decreasing ACh and AChR levels and increasing AChE. Needling muscle fibers outside of MTrSs in the DN-nM group were less effective than in the DN-M group. This result indicated the importance of dry needling MTrS to elicit LTRs. A strong stimulation at MTrP causes a strong spinal cord reflex that breaks the cycle of the MTrP circuit in the spinal cord [29], whereas dry needling non-MTrS regions may induce weak changes in the spinal cord reflex. Dry needling can relieve pain in MTrPs possibly by reducing the amplitudes and frequencies of EPN and EPS; by decreasing the excessive release of ACh and AChR; and by increasing AChE in the endplate of MTrPs. Further studies on the neural pathway and dry needling treatment for a long period are necessary.

## 5. Conclusions

Dry needling at the exact MTrSs is more effective than dry needling at non-MTrSs, which is reflected in parameters of SEA, ACh, AChR, and AChE. Although dry needling MTrS twice cannot completely eliminate the EMG activities, it can decrease SEA activities. This result implies that dry needling at MTrSs should be conducted more than twice to complete pain treatment.

## Figures and Tables

**Table 1:** Changes in the amplitudes and frequencies of EPN and EPS before and after dry needling.

Groups	Parameters	EPN		EPS	
		BDN	ADN	BDN	ADN
BC (*n* = 12)	Amplitude (*µ*V)	4.38 ± 1.57	4.35 ± 1.19	/	/
	Frequency (Hz/s)	/	/	/	/
MC (*n* = 12)	Amplitude (*µ*V)	29.62 ± 3.71	30.81 ± 3.11	188.12 ± 43.06	244.79 ± 55.17
	Frequency (Hz/s)	10.25 ± 3.60	11.33 ± 2.25	67.83 ± 21.23	72.75 ± 19.42
DN-M (*n* = 12)	Amplitude (*µ*V)	27.39 ± 7.25	13.42 ± 0.73^*∗∗*##^	212.50 ± 47.21	142.08 ± 31.76^*∗∗*##^
	Frequency (Hz/min)	10.92 ± 4.80	6.75 ± 1.95^*∗∗*##^	63.58 ± 18.79	30.83 ± 8.27^*∗∗*##^
DN-nM (*n* = 12)	Amplitude (*µ*V)	28.27 ± 6.28	23.06 ± 4.15^*∗∗*^	219.79 ± 56.09	230.17 ± 87.50
	Frequency (Hz/min)	11.67 ± 3.77	9.71 ± 3.01	69.75 ± 23.94	70.33 ± 21.17

*Note*. ^*∗∗*^*p* < 0.01 indicates a significant difference in amplitudes or frequencies among groups. ^##^*p* < 0.01 indicates a significant difference in amplitudes or frequencies compared with before and after dry needling treatment. BC: blank control, MC: MTrSs control, DN-M: dry needling at MTrSs, and DN-nM: dry needling at non-MTrSs; EPN: endplate noise; EPS: endplate spikes; BDN: before dry needling; ADN: after dry needling.

## References

[1] Simons D. G., Travell J. G., Simons L. S. (1999). *Travell and Simons’ Myofascial Pain and Dysfunction: The Trigger Point Manual*.

[2] Hong C.-Z. (1994). Lidocaine injection versus dry needling to myofascial trigger point: the importance of the local twitch response. *American Journal of Physical Medicine and Rehabilitation*.

[3] Hong C.-Z., Simons D. G. (1998). Pathophysiologic and electrophysiologic mechanisms of myofascial trigger points. *Archives of Physical Medicine and Rehabilitation*.

[4] Hong C.-Z., Torigoe Y. (1994). Electrophysiological characteristics of localized twitch responses in responsive taut bands of rabbit skeletal muscle fibers. *Journal of Musculoskeletal Pain*.

[5] Huang Q.-M., Lv J.-J., Ruanshi Q.-M., Liu L. (2015). Spontaneous electrical activities at myofascial trigger points at different stages of recovery from injury in a rat model. *Acupuncture in Medicine*.

[6] Huang Q.-M., Ye G., Zhao Z.-Y., Lv J.-J., Tang L. (2013). Myoelectrical activity and muscle morphology in a rat model of myofascial trigger points induced by blunt trauma to the vastus medialis. *Acupuncture in Medicine*.

[7] Simons D. G., Hong C.-Z., Simons L. S. (1995). Prevalence of spontaneous electrical activity at trigger spots and at control sites in rabbit skeletal muscle. *Journal of Musculoskeletal Pain*.

[8] Gerwin R. D., Dommerholt J., Shah J. P. (2004). An expansion of Simons' integrated hypothesis of trigger point formation. *Current Pain and Headache Reports*.

[9] Liley A. W. (1956). An investigation of spontaneous activity at the neuromuscular junction of the rat. *The Journal of Physiology*.

[10] Hubbard D. R., Berkoff G. M. (1993). Myofascial trigger points show spontaneous needle EMG activity. *Spine*.

[11] Simons D. G. (2001). Do endplate noise and spikes arise from normal motor endplates?. *The American Journal of Physical Medicine and Rehabilitation*.

[12] Rai S., Ranjan V., Misra D., Panjwani S. (2016). Management of myofascial pain by therapeutic ultrasound and transcutaneous electrical nerve stimulation: a comparative study. *European Journal of Dentistry*.

[13] Segura-Ortí E., Prades-Vergara S., Manzaneda-Piña L., Valero-Martínez R., Polo-Traverso J. A. (2016). Trigger point dry needling versus strain-counterstrain technique for upper trapezius myofascial trigger points: a randomised controlled trial. *Acupuncture in Medicine*.

[14] Fernández-Carnero J., La Touche R., Ortega-Santiago R. (2010). Short-term effects of dry needling of active myofascial trigger points in the masseter muscle in patients with temporomandibular disorders. *Journal of Orofacial Pain*.

[15] Liu L., Huang Q.-M., Liu Q.-G. (2015). Effectiveness of dry needling for myofascial trigger points associated with neck and shoulder pain: a systematic review and meta-analysis. *Archives of Physical Medicine and Rehabilitation*.

[16] Chen J.-T., Chung K.-C., Hou C.-R., Kuan T.-S., Chen S.-M., Hong C.-Z. (2001). Inhibitory effect of dry needling on the spontaneous electrical activity recorded from myofascial trigger spots of rabbit skeletal muscle. *American Journal of Physical Medicine and Rehabilitation*.

[17] Hsieh Y.-L., Yang C.-C., Liu S.-Y., Chou L.-W., Hong C.-Z. (2014). Remote dose-dependent effects of dry needling at distant myofascial trigger spots of rabbit skeletal muscles on reduction of substance P levels of proximal muscle and spinal cords. *BioMed Research International*.

[18] Zimmermann M. (1986). Ethical considerations in relation to pain in animal experimentation. *Acta Physiologica Scandinavica. Supplementum*.

[19] Zimmermann M. (1983). Ethical guidelines for investigations of experimental pain in conscious animals. *Pain*.

[20] Kuan T.-S., Chen J. T., Chen S. M., Chien C. H., Hong C. Z. (2002). Effect of botulinum toxin on endplate noise in myofascial trigger spots of rabbit skeletal muscle. *American Journal of Physical Medicine and Rehabilitation*.

[21] Hsieh Y.-L., Yang S.-A., Yang C.-C., Chou L.-W. (2012). Dry needling at myofascial trigger spots of rabbit skeletal muscles modulates the biochemicals associated with pain, inflammation, and hypoxia. *Evidence-Based Complementary and Alternative Medicine*.

[22] Chu J. (1995). Dry needling (intramuscular stimulation) in myofascial pain related to lumbosacral radiculopathy. *European Journal of Physical Medicine and Rehabilitation*.

[23] Martyn J. A. J., White D. A., Gronert G. A., Jaffe R. S., Ward J. M. (1992). Up-and-down regulation of skeletal muscle acetylcholine receptors: effects on neuromuscular blockers. *Anesthesiology*.

[24] Brett R. S., Schmidt J. H., Gage J. S., Schartel S. A., Poppers P. J. (1987). Measurement of acetylcholine receptor concentration in skeletal muscle from a patient with multiple sclerosis and resistance to atracurium. *Anesthesiology*.

[25] Hogue C. W., Itani M. S., Martyn J. A. J. (1990). Resistance to d-tubocurarine in lower motor neuron injury is related to increased acetylcholine receptors at the neuromuscular junction. *Anesthesiology*.

[26] Kim C., Martyn J., Fuke N. (1988). Burn injury to trunk of rat causes denervation-like responses in the gastrocnemius muscle. *Journal of Applied Physiology*.

[27] Liu X., Zhu B., Zhang S. (1986). Relationship between electroacupuncture analgesia and descending pain inhibitory mechanism of nucleus raphe magnus. *Pain*.

[28] Hong C. Z. (1994). Considerations and recommendations regarding myofascial trigger point injection. *Journal of Musculoskeletal Pain*.

[29] Hong C.-Z. (2006). Treatment of myofascial pain syndrome. *Current Pain and Headache Reports*.

